# Influence of Hypoxic Condition on Cytotoxicity, Cellular Migration, and Osteogenic Differentiation Potential of Aged Periodontal Ligament Cells

**DOI:** 10.1055/s-0044-1786844

**Published:** 2024-05-17

**Authors:** Sukrit Jaiklaew, Kallapat Tansriratanawong

**Affiliations:** 1 Department of Oral Medicine and Periodontology, Faculty of Dentistry, Mahidol University, Bangkok, Thailand

**Keywords:** periodontal ligament, cell hypoxia, cell migration assays, osteogenesis, cell differentiation

## Abstract

**Objective** This study aimed to investigate and compare the influence of hypoxic conditions on cytotoxicity, cellular migration, and osteogenic differentiation of aged periodontal ligament (PDL) cells.

**Materials and Methods** Isolated human PDL cells from aged and young subjects were cultured under hypoxic conditions, which were treated with hydrogen peroxide (H _2_ O _2_ ) (0, 25, 50, 100, 200, and 500 µM). To assess cytotoxicity, lactate dehydrogenase release was determined by the optical density at 490 nm, and the percentage of cell death was calculated. An *in vitro* wound healing assay was performed over 24 to 48 hours for cellular migration determination. Osteogenic differentiation was determined by alizarin red staining and osteogenic gene expression, including the expression of runt-related transcription factor 2 (RUNX2), alkaline phosphatase (ALP), and osteopontin (OPN).

**Results** There was a significant difference in the percentage of cell death with high hypoxic condition (200 and 500 µM) compared to low hypoxic conditions on both day 1 and 2. The highest cellular migration was depicted at 50 µM in both young and aged groups of the *in vitro* wound healing assay. Osteogenic gene expression of RUNX2 in the aged group was increased at 25 and 50 µM hypoxic condition at day 7, but the expression was gradually decreased after 14 days. On the contrary, the expression of ALP and OPN in the aged group was increased at day 14. Only OPN had been found to be statistically significantly different when compared with gene expression at day 7 and 14 ( *p* < 0.05). The results showed no statistically significant differences when compared with the young and aged groups in all genes and all concentrations.

**Conclusion** The concentration of low hypoxic condition (25–50 µM) was proposed to promote cell viability, cellular migration, and osteogenic differentiation in aged PDL cells. We suggested that the potential of aged PDL cells for use in cell therapy for periodontal regeneration might possibly be similar to that of young PDL cells.

## Introduction

Periodontal disease is a chronic inflammatory disease in which bacteria infect, invade, and destroy the periodontium, including the gingiva, cementum, periodontal ligament (PDL), and alveolar bone. It is initiated by periodontal pathogenic bacterial stimulation from dental biofilms that deposit on the surface of teeth, implants, or prostheses and result in an imbalance of the environment within the biofilms, and eventually cause the loss of alveolar bone support. Disease progression is not only dependent on the pathogenic bacteria but also modified by host susceptibility and the immune response. 1 2 The immune response can be altered and modified by several factors, such as genetics, environment, behavior, and aging. 2 Various biological and physiological changes in immune responses have been suggested to be age-related, including increased serum C-reactive protein levels, changes in inflammatory mediators, and reduced levels of serum folate, which is a protective factor against chronic inflammatory diseases. 3 4 5

Aging is the biological process of growing older and has been suggested to involve the accumulation of cellular damage over time. During the aging process, the periodontium changes physically, biologically, and functionally, which leads to enhanced bacterial invasion, and the progression of disease may be faster with increasing age, a low rate of cell turnover, susceptibility to inflammation, and impaired dexterity that leads to poor biofilm control. 5 6 Thus, age has been suggested to be a risk determinant for periodontal disease, with an odds ratio of 1.05 to 2.29. 7 8 Nevertheless, it has been established that these age-related factors of periodontal disease have negligible effects on an individual's responsiveness to periodontal treatment if the patient has good biofilms control. 9 Compared with younger patients, aged patients with periodontitis showed no difference in the clinical response to nonsurgical treatment. 10 Regarding cell-based therapy for periodontal regeneration, the differentiation potential of osteoblastic progenitor cells derived from aged individuals is highly variable, and it remains unclear whether aged patients have worse regenerative results or are similar to those of young patients. 11 12 13

One possible mechanism drawn from the mitochondrial theory of aging is because mitochondria can induce oxidative damage, it can result in reactive oxygen species (ROS) production. Overabundant ROS has harmful effects on mitochondrial function and causes cumulative damage; therefore, cells try to compensate by producing antioxidants. If the damage is overwhelming, the mitochondria are not able to supply enough energy and generate oxygen-deprived conditions, which contribute to the aging process and lead to hypoxia. 14 However, low concentrations of exogenous H _2_ O _2_ and low levels of ROS contribute to hypoxic conditions in mesenchymal stem cells (MSCs) and have been reported to promote MSC proliferation and migration via the extracellular-signal-regulated kinase 1/2 and Jun-1/2 pathways. 15 16 17 18 Additionally, growth factors such as platelet-derived growth factor-BB and vascular endothelial growth factor (VEGF) were suggested to be inducible and to exhibit enhanced expression under low-level ROS conditions, which also promotes the proliferation and migration of MSCs. 19 20 21 The VEGF gene is highly expressed and enhances angiogenesis under hypoxic conditions via hypoxia-inducible factor-1 alpha (HIF-1α) in PDL fibroblasts. 22 23 Therefore, HIF was suggested to be a linking factor that may play a key role in inducing the overexpression of angiogenesis-related factors and contribute to osteogenic differentiation in PDL cells. 24

Evidence on low-level hypoxic conditions and their effects in PDL cells derived from aged subjects is limited. PDL cells were selected as candidate cells due to their progenitor or stem cell-like properties, including self-renewal, clonogenicity, multilineage differentiation, ease of harvest, and they may be a useful cell source for periodontal regeneration. 25 Moreover, although the prevalence of periodontal disease is increased in aging subjects due to the accumulation of damage, disease progression, and impaired periodontal wound healing in aged cells, the disease does not always progress. We structured the null hypothesis stating that the influence of hypoxic condition on cytotoxicity, cellular proliferation, cellular migration, and osteogenic differentiation of aged PDL cells does not differ from that of young PDL cells. Conversely, the alternative hypothesis is that a low level of hypoxia may promote periodontal regeneration in aged patients. Therefore, this study aimed to investigate and compare the influence of hypoxic conditions on cytotoxicity, cellular migration, and osteogenic differentiation of aged PDL cells.

## Materials and Methods

### Study Populations

All subjects who were enrolled in this study were asked to provide informed consent. The inclusion criteria for the aged group were subjects who were 60 years old or older. For comparison, the young group included subjects who were less than 30 years old. All subjects were clinically healthy or had well-controlled systemic diseases. The exclusion criteria were subjects who had uncontrolled systemic diseases that caused impaired healing and women who were pregnant or lactating. All studies were conducted according to the guidelines of the research ethics committee of Mahidol University, Bangkok, Thailand (COE.No. MU-DT/PY-IRB 2021/024.3007).

### Human PDL Cell Isolation and Culture

Intact periodontal teeth, which demonstrated absence of bleeding on probing (probing depth ≤ 4 mm) and had noncarious lesions on the root surface, were extracted from 10 subjects in each aged and young groups for orthodontic reasons, prosthodontic reasons, endodontic reasons, removal of wisdom teeth, or simple extraction at the Oral and Maxillofacial Surgery Clinic, Mahidol University, Bangkok, Thailand. The teeth were randomly used for at least three samples in each group in all experiments. The teeth were washed twice with phosphate-buffered saline (PBS), and PDL tissues were scraped and isolated from the middle one-third of the noncarious root surfaces with a sterile scalpel. The tissues were minced into small pieces and further isolated within 24 hours by the outgrowth method. The isolated PDL cells were placed into 60-mm dishes (Corning, New York, United States) with 3 mL of culture medium that was prepared from Dulbecco's modified Eagle's medium containing 15% fetal bovine serum, 0.05% glutamine (Gibco BRL, Carlsbad, California, United States), 0.5% penicillin/streptomycin (Gibco BRL), and 0.1% amphotericin B (Gibco BRL) as growth medium (GM) and cultured at 37°C in a 20% O _2_ and 5% CO _2_ atmosphere.

Fibroblast-like cells, which were derived from PDL tissues and migrated from the tissue fragments, were isolated and defined as primary PDL cells. Colony formation of PDL cells was investigated to characterize progenitor potential. After the PDL cells reached approximately 80 to 90% confluence, the cells were subcultured at a dilution of 1:3 and were defined as the 1st passage. The human PDL cell line was purchased, cultured according to the manufacturer's instructions, and used as a control (HPDLF, Catalog Number: 2630, ScienCell, California, United States). The 3rd to 7th passages of aged and young PDL cells were used in all experiments.

### Senescence Assay

Senescence detection was performed with a senescent cell histochemical staining kit (Sigma-Aldrich, Missouri, United States) to evaluate and compare the primary senescence characteristics of the aged and young groups at the 3rd passage. The staining mixture was prepared by mixing 1 mL of staining solution, 125 μL of reagent B, 125 µL of reagent C, 0.25 mL of X-gal solution, and 8.5 mL of water. The cells from each group were seeded at a density of 5 × 10 ^4^ cells/1 mL/well in a 24-well plate with normal GM for 24 hours. Then, the GM was removed and the cells were washed twice with PBS. The fixation buffer was added to each well and incubated for 7 minutes at room temperature (25°C). Then, the cells were washed three times with PBS and a staining mixture was added to each well. The plate was incubated at 37°C without CO _2_ for 24 hours (Thermo Fisher Scientific Inc., United States). The image of each well at 10× magnification under microscope was captured. Each picture was named. The name of each picture was randomly chosen for 10 images for each group and the blue-stained cells (senescent cells) were counted and calculated from each group with the following formula:



### Proliferation Assay

Cellular proliferation was examined by 3-(4,5-dimethylthiazol-2,5-diphenyl tetrazolium bromide (MTT; PanReac Appli Chem, Germany) for proliferation assay among control, aged, and young group. The 5 × 10 ^3^ cells suspension/well were seeded in 96-well plates (NUNC) and incubated for 24 hours. Supernatant was replaced by the fresh GM and cells from three groups were incubated under the same condition at 24 to 48 hours. Each well was added with 20 µL of prewarmed (37°C) MTT solution and cultured for 2 hours. Dimethyl sulfoxide was used for inhibiting the reaction by adding to each well at 37°C for 30 minutes. The optical density (OD) of solution was measured at 570 nm of wavelength by enzyme-linked immunosorbent assay (ELISA) plate reader (Epoch, BioTek Instruments, Inc., United States).

### Preparation of H _2_ O _2_ for Hypoxic Cell Culture Conditions

The stock solution was prepared from a 10-mM H _2_ O _2_ solution in sterile distilled water. Then, the stock solution was diluted in GM until it reached the final concentration, which was 25, 50, 100, 200, or 500 µM for all experiments. For the hypoxic condition, PDL cells were seeded in 24-well plates at an initial density of 2 × 10 ^5^ cells/mL for 24 hours, and GM with 25, 50, 100, 200, and 500 µM H _2_ O _2_ was added to each well. In contrast, the normoxic condition was defined as PDL cells seeded in GM at the same cell density in the absence of H _2_ O _2_ . PDL cells from all conditions were cultured at 37°C in a 20% O _2_ and 5% CO _2_ atmosphere.

### Cytotoxicity Assay

Cytotoxicity was measured by the release of the enzyme lactate dehydrogenase (LDH) in the GM (Roche, Mannheim, Germany). LDH is a stable cytoplasmic enzyme that is found in all cells and is rapidly released into the cell culture supernatant when the plasma membrane is damaged. PDL cells were seeded in 24-well plates at an initial density of 2 × 10 ^5^ cells/mL for 24 hours, and then the supernatant was pooled and collected into a 15-mL centrifuge tube (Corning, New York, United States) for each group. The 50-μL of supernatant from each group at all concentrations was transferred to a 96-well plate in triplicate. The normoxic GM was used as a negative control, and the wells of the PDL cell line, which were supplemented with 5 μL of lysis buffer (2% Triton X-100 solution) and incubated for 15 minutes, were used as a positive control. Then, 50 μL of the LDH assay reagent was added to the supernatants as a substrate. The reagents were mixed by gentle shaking for 30 seconds. The plate was incubated at 37°C in a 20% O _2_ and 5% CO _2_ atmosphere for 10 minutes in the incubator (Thermo Fisher Scientific Inc.). Then, 100 μL of stop solution (hydrochloric acid 1 N) was added to each well, and the reagent was mixed by shaking for 30 seconds. The absorbance of each well was measured at 490 nm (setting the background absorbance at 690 nm) within 1 hour. Cytotoxicity, which was induced by H _2_ O _2_ , was observed. The percentage of cell death (% cytotoxicity) was calculated from the following equation:



### Cellular Migration ( *In* *Vitro* Wound Healing Assay)

The *in vitro* wound healing assay was performed in triplicate. PDL cells in each group were seeded in 24-well plates at a density of 2 × 10 ^5^ cells/well and cultured as a monolayer for 1 to 2 days until the cells reached 90 to 100% confluence. PDL cells were scratched vertically with a 1,000-µL pipette tip to create an artificial wound. For the baseline area (day 0), the medium was removed immediately after scratching. The cells were washed twice with PBS and fixed with 100% methanol for 10 minutes. After fixation, the PDL cells were washed twice with PBS to remove residual methanol, stained with 10% Giemsa (Merck, Missouri, United States) for 10 minutes and finally rinsed with water. The area of the artificial wound was assessed under an inverted microscope (Olympus CKX53, Shinjuku, Japan) using 4× magnification. The artificially wounded PDL cell cultures were subjected to normoxic and hypoxic conditions for 24 and 48 hours, respectively. Afterward, the artificially wounded cells were washed twice with PBS, fixed with 100% for 10 minutes, washed twice with PBS, and stained with 10% Giemsa. The areas of day 0, day 1, and day 2 groups were measured, and the surface area was estimated with ImageJ (National Institutes of Health, United States) and calculated with the following formula:



### Osteogenic Differentiation

PDL cells were seeded at a density of 2 × 10 ^5^ cells/well in 6-well plates with normal GM until they reached 80 to 90% confluence. Then, PDL cell cultures were subjected to normoxic and hypoxic osteogenic induction media, which included 100 nM dexamethasone (Sigma-Aldrich), 50 µM ascorbic acid (Sigma-Aldrich), and 10 mM β-glycerophosphate (Sigma-Aldrich) in GM. Osteogenic differentiation of the aged and young groups was detected by mineralization assays and gene expression at 7 and 14 days.

### Alizarin Red Staining

Alizarin red staining (ARS) solution was prepared by dissolving 3 g alizarin red (Sigma-Aldrich) in 100 mL distilled water and gently mixing. The pH was adjusted with hydrochloric acid (RCI Labscan, Ireland) until it was approximately 4.1 to 4.3. PDL cells with osteogenic induction were washed twice with PBS (VWR, France) and fixed with 10% buffer formalin (Loba Chemie, India) for 10 minutes. ARS solution was added to each well and incubated at room temperature for 45 minutes protected from light. After the ARS solution was aspirated, the cells were washed with distilled water several times and inspected with a phase contrast microscope. For the quantification assay, 800 µL of 10% acetic acid (RCI Labscan) was added to osteogenic induction in each well and incubated at room temperature for 30 minutes. Then, the cells were transferred to a 1.5-mL microcentrifuge tube and vortexed for 30 seconds (Vortex2, Scientific Industries, New York, United States). The solution with cells was heated at 85°C for 10 minutes, incubated on ice for 5 minutes, and centrifuged at 20,000 × *g* for 15 minutes (Hettich Universal, Singapore). Then, 500 µL of the supernatant was transferred to a new tube. Next, 200 µL of 10% ammonium hydroxide (Loba Chemie) was added to neutralize the acid. A 150-µL aliquot of the sample was transferred into a 96-well plate, and the absorbance was read at 405 nm in ELISA plate reader (Epoch, BioTek Instruments, Inc.). The assay was performed for 7 and 14 days. The ARS standard solution was prepared from 100 µL of 40 mM ARS solution and 900 µL of distilled water and adjusted pH until 4.1 to 4.3 with 10% ammonium hydroxide and 10% acetic acid to make 1 mL of 4 mM ARS. The solution was mixed well in 1.5 mL microcentrifuge tubes. Then, 500 µL of standard solution was added to each tube; 500 µL of 4 mM ARS solution was added to obtain 2 mM ARS standard in the first tube and 500 µL of 2 mM ARS solution was transferred to the next tube to obtain 1 mM ARS solution. The procedure was repeated, and the solution serially diluted to 2, 1, 0.5, 0.25, 0.125, 0.0625, and 0.0313 mM and blank. A total of 150 µL of each concentration was transferred to a 96-well plate in triplicate. The absorbance at 405 nm was read. These absorbance values were plotted as points to illustrate the relationship between concentration and absorbance. A quantification standard curve was generated from these points and the resulting equation was utilized for quantification purposes.

### Real-Time Reverse Transcriptase Polymerase Chain Reaction

Osteogenic markers in differentiated cells were evaluated with real-time reverse transcriptase polymerase chain reaction (RT-PCR) to compare gene expression between day 7 and day 14 for aged and young groups. Osteogenic induction and noninduction underwent osteogenic differentiation under normoxic and hypoxic conditions. The markers were HIF-1α, runt-related transcription factor 2 (RUNX2), alkaline phosphatase (ALP), and osteopontin (OPN), and the primer sequences are presented in Supplementary Table S1 (available in the online version). The levels of β-actin expression were used as an endogenous control. The ribonucleic acid (RNA) from the samples was prepared with FavorPrep (Favogen Biotech Corp., Ping-Tung, Taiwan) and determined from the absorbance at 260/280 nm (NanoDrop, Thermo Fisher Scientific, Massachusetts, United States). Then, 500 ng of RNA was used along with forward and reverse PCR primers to amplify HIF-1α and osteogenesis-related genes (CFX96 touch real-time PCR detection system, Bio-Rad, California, United States). Real-time RT-PCR was performed with qPCRBIO SyGreen 1-step Go Lo-ROX (PCR Biosystems, London, United Kingdom). Reactions were done with 2× qPCRBIO SyGreen 1-step Mix, 20× RTase Go, primers, and distilled water. Reverse transcription was performed for 10 minutes at 45°C. The polymerase activation was activated at 95°C for 2 minutes. The amplification consisted of 50 cycles of 95°C for 5 seconds, annealing at 56 to 60°C for 30 seconds, and a final extension step at 95°C for 15 seconds. The assay was performed in duplicate and data were derived from quantitative data and compared with endogenous genes. The result of real-time RT-PCR was shown as increasing fluorescence signals and was measured as the Ct value. The relative fold change in gene expression was calculated with the 2 ^-∆∆CT^ method.

### Statistical Analysis

All data were analyzed for a normal distribution with the Shapiro–Wilk test. Cell cytotoxicity, cellular migration assay, quantification of ARS, and real-time RT-PCR were presented as the median, 25th percentile, and 75th percentile. The senescence data was illustrated as the mean ± standard deviation. Kruskal‒Wallis test was used to compare the percentage of cell death and cellular migration assay among groups. Cellular proliferation was analyzed by one-way analysis of variance for comparing means among control, young, and aged group. The Mann–Whitney *U* test was used to compare the concentration of ARS and relative gene expression between two groups. Wilcoxon signed rank test was used to compare the concentration of ARS and relative gene expression between 7 and 14 days. Friedman test was used to compare the percentage of cell death, percentage cellular migration, concentration of ARS, and relative gene expression among concentrations. All possible pairwise comparisons were performed using Bonferroni adjustment. The cytotoxicity assay, cellular migration assay, and quantification of ARS were performed in triplicate, while real-time RT-PCR was performed in duplicate. SPSS program ver. 28 (SPSS Inc., Chicago, Illinois, United States) was used for the analyses. A *p* -value of < 0.05 was considered statistically significant.

## Results

### Senescent Characteristics of PDL Cells

**Fig. 1:**
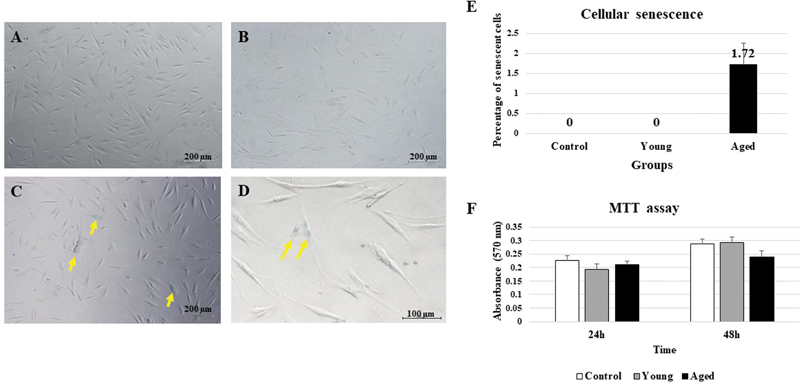
Senescent assay. ( **A** ) Image from phase contrast microscope of control (periodontal ligament [PDL] cell line), ( **B** ) young group, and ( **C** ) aged group. ( **A** – **C** ) 4× magnification and scale bar = 200 µm. ( **D** ) Aged group with 10× magnification. Scale bar = 100 µm. ( **E** ) Percentage of senescent cells in passage 3. ( **F** ) Cellular proliferation of control, young, and aged groups ( *n* = 10).

Senescent staining was performed for the PDL cell line (control), the young, and aged groups ( Fig. 1A–C ), respectively (magnification 4 × ). The higher magnification (10 × ) of the aged group is depicted in Fig. 1D . Βeta-galactosidase is a marker of senescent cells and displayed in blue in the nucleus ( Fig. 1C, D ). The results showed that cellular senescence was exclusively observed in the aged group, while senescence was not observed in the control and young groups. The percentages of stained cells in the control, young, and aged groups were 0, 0, and 1.72%, respectively ( Fig 1E ).

### Cellular Proliferation of PDL Cells

PDL cells of young and aged groups demonstrated lower proliferation potential when compared to the control group at 24 hours. There were no statistically significant differences among the three group at 24 hours ( *p* > 0.05, Fig. 1F ). By time-dependence at 48 hours, the young group showed the highest proliferation rate when compared to the control and aged group, but there were no statistically significant differences among the three groups ( *p* > 0.05, Fig. 1F ).

### Increasing Amounts of Cell Death at High Concentrations

**Fig. 2:**
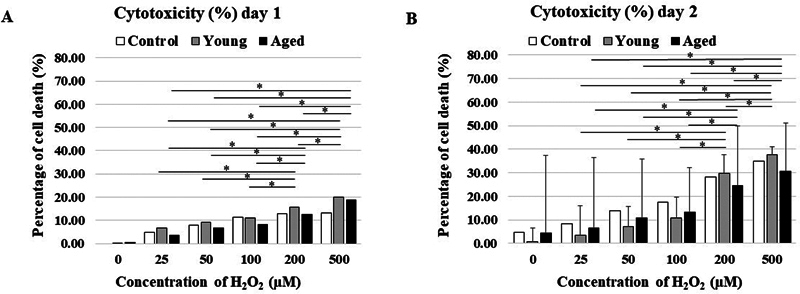
Comparing each concentration of cytotoxic assay. ( **A** ) Chart showed percentage of cytotoxicity on day 1. ( **B** ) Chart showed percentage of cell death on day 2. The differences in each group were significant as shown by star (*). Concentration of 200 and 500 µM were significantly different from other concentrations ( *p* < 0.05) for both of day 1 and day 2 ( *n* = 3).

Cytotoxicity was demonstrated by the percentage of cell death in the LDH assay at 24 hours (day 1) and 48 hours (day 2). Cytotoxicity from 25 to 500 µM of all groups was trended to gradual increase by H _2_ O _2_ concentration dependence. Statistically significant differences were demonstrated in the percentage of cell death with high concentrations (200 and 500 µM) compared to low concentration on day 1 and 2 ( Fig. 2A , B, *p* < 0.05).

### Similar Wound Closure in the Aged and Young Groups Under Low-Level Hypoxic Conditions

**Fig. 3:**
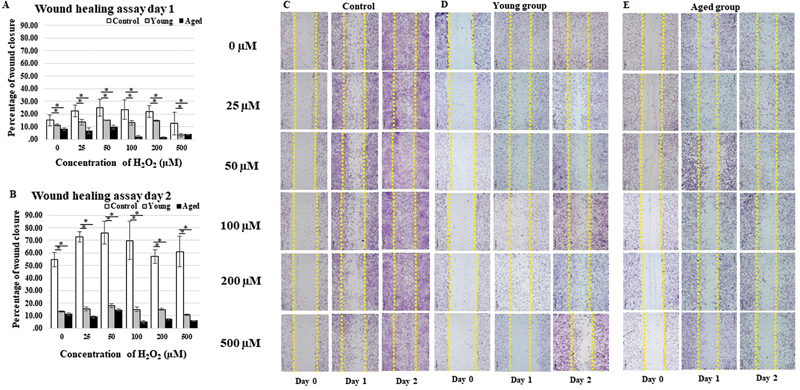
For cellular migration in each concentration (0, 25, 50, 100, 200, and 500 µM) and day: ( **A** ) day 1 and ( **B** ) day 2. Control had a significant difference compared to the aged and young groups at all concentrations on day 1 and 2 ( *p* < 0.05). The differences were significant as shown by star (*). Pictures depicted *in vitro* wound healing staining with Giemsa ( **C** ) control, ( **D** ) young, and ( **E** ) aged group (scale bar = 200 µm, *n* = 10).

An *in vitro* wound healing assay was performed to analyze the cellular migration of PDL cells after treatment with H _2_ O _2_ in the aged and young groups. The aged and young groups exhibited similar cellular migration rates of all concentrations on day 1 and 2 ( Fig. 3A, B ). The trend of cellular migration was decreasing when higher concentrations were used in all groups. Interestingly, the control group demonstrated strong cellular migration even when H _2_ O _2_ was applied, and the percentages varied from 54.78 to 78.55% on day 2, with a significant difference compared to either the aged or young groups at all concentrations ( *p* < 0.05 Fig. 3A–C ). However, there was no statistically significant difference between aged and young groups.

### Mineralization Determined by Alizarin Red Staining

**Fig. 4:**
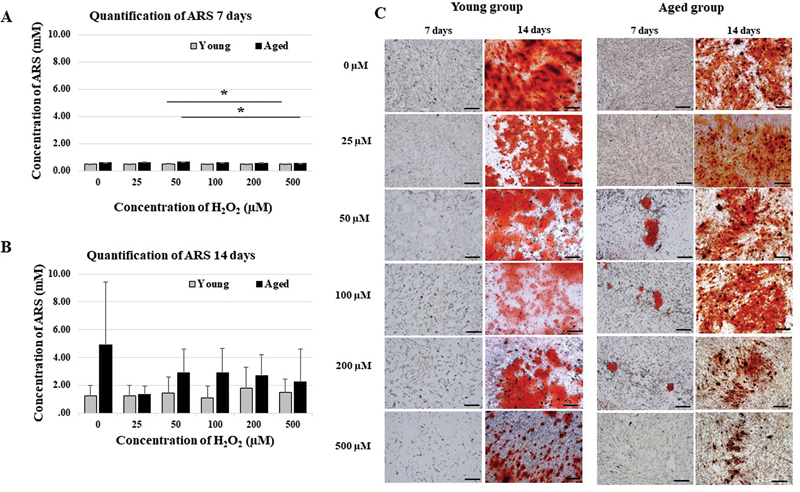
Quantification of alizarin red staining (ARS). ( **A** ) At 7 days, the concentration of ARS (mM) for 50 µM concentration of both aged and young groups were significantly different when compared to 500 µM ( *p* = 0.016 and 0.016, respectively). ( **B** ) At 14 days, the concentration of ARS for all concentrations were not significant ( *p* ≥ 0.05, scale bar = 200 µm, *n* = 3).

The standard curve of ARS is plotted according to the equation ARS = 0.751 (OD 405 nm) + 0.0589. Quantification and staining of ARS were performed as shown in Fig. 4A–C . On day 7, 50 µM in the aged and young groups significantly differed from 500 µM in the aged and young groups ( *p* = 0.016 and 0.016, respectively, Fig. 4A ). None of the concentrations of ARS were significantly different between the aged and young groups at day 14 ( *p* ≥ 0.05, Fig. 4B ). Although there were no statistically significant difference at all concentrations, interestingly, day 14 had higher mineralization in the aged group than in the young group ( Fig. 4B ).

### Gene Expression of HIF-1α and Osteogenic Genes

**Fig. 5:**
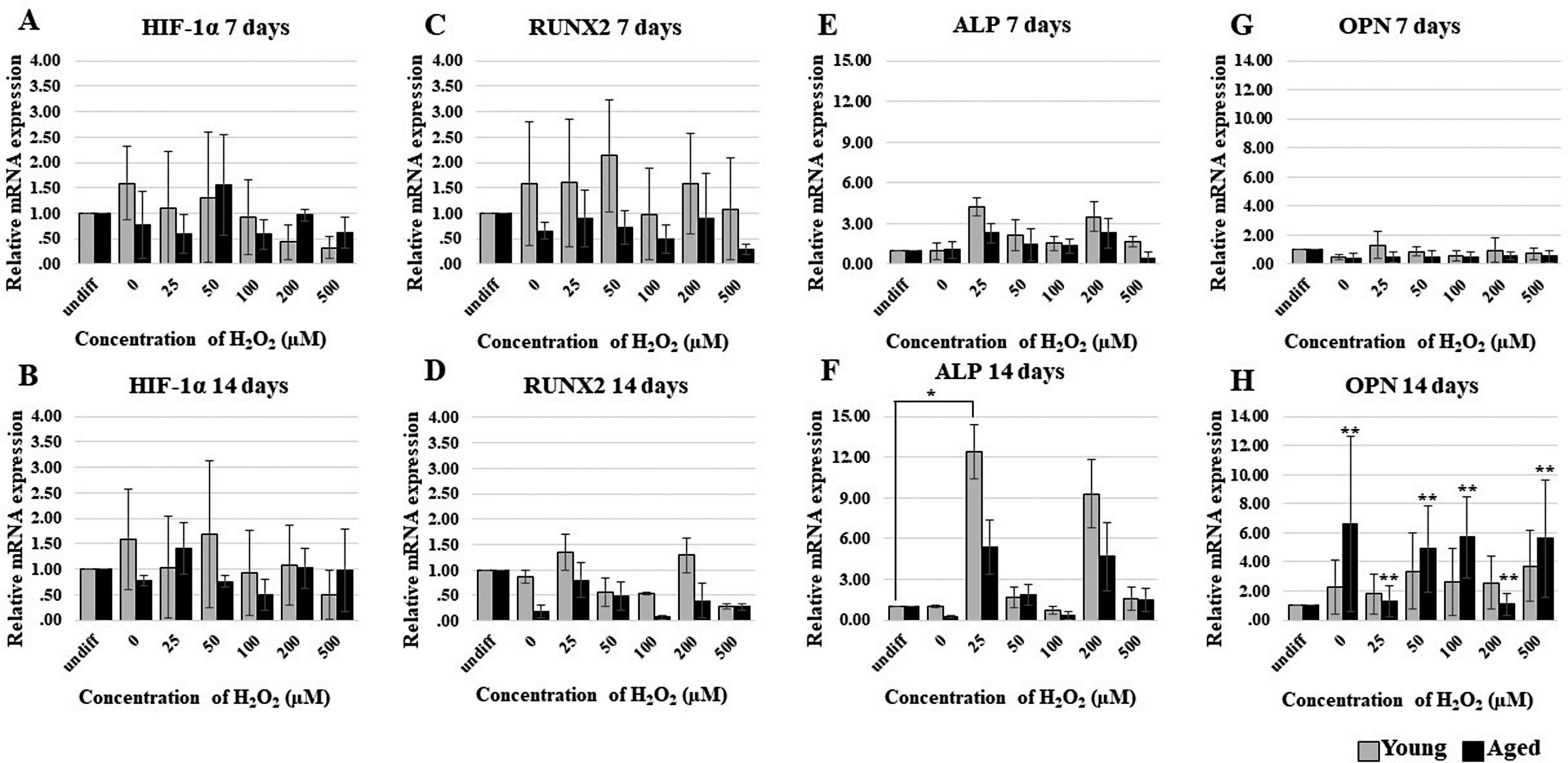
Gene expression of ( **A** ) hypoxia-inducible factor-1 alpha (HIF-1α) on day 7, ( **B** ) HIF-1α on day 14, ( **C** ) runt-related transcription factor 2 (RUNX2) on day 7, and ( **D** ) RUNX2 on day 14. The expressions of young and aged group were not different for all concentration ( *p* ≥ 0.05, *n* = 3). ( **E** ) Alkaline phosphatase (ALP) on day 7 and ( **F** ) ALP on day 14. The expression of ALP at concentration 25 and 0 µM of the young group was statistically significantly different ( *p* = 0.037, *n* = 3). ( **G** ) Osteopontin (OPN) on day 7 and ( **H** ) OPN on day 14. The OPN gene expression between 7 and 14 days was statistically significantly different ( *p* < 0.05, *n* = 3).

Gene expression of the HIF-1α gene is displayed in Fig. 5A, B . Interestingly, the highest expression was observed at a concentration of 50 µM in the aged group on day 7 and at a concentration of 25 µM in the aged group on day 14, respectively ( Fig. 5A, B ), and in the young group at the same concentrations (50 µM) on day 14 ( Fig. 5B ), but they were no statistically significant differences when compared to other groups and concentrations.

When the gene expression of osteogenic genes was examined ( Fig. 5C–H ). The RUNX2 gene showed the highest expression at a concentration of 50 µM on day 7 and at a concentration of 25 µM on day 14, and there was no significant difference in RUNX2 gene expression between the aged and young groups at all concentrations ( Fig. 5C, D ). At a concentration of 25 µM on day 14 of the ALP gene, a statistically significant difference was demonstrated in the young group when compared to undifferentiated cells ( *p* = 0.037, Fig. 5F ). For OPN, there were significant differences of aged group in all concentrations when comparing day 7 with day 14 ( *p* < 0.05, Fig. 5G, H ); however, there was no significant difference between the aged and young groups at all concentrations.

## Discussion

Low-level hypoxic conditions have been studied as transient mechanisms of tissue repair and regeneration. HIF-1α, a hypoxia-related gene, mediates as an intermediary involved in proliferation, cellular migration, and osteogenic differentiation. The hypoxic pathways can upregulate the VEGF expression via HIF-1α, which enhances angiogenesis and reoxygenation of this hypoxic environment. 19 20 21 22 23 24 Moreover, several pluripotency reprogramming factors, which induce lineage differentiations and commit in embryonic development such as Sox2, Oct4, and Nanog, have been suggested that they can exhibit transiently upregulation in response to ischemic sites in the induced pluripotent stem cell. 26 27 Therefore, low-level hypoxic conditions might play a role in stemness maintenance, proliferation, and differentiation.

With low concentrations of 25 and 50 µM, aged PDL cells demonstrated cytotoxicity, cellular migration, and osteogenic differentiation similar to those of the young group. However, high concentrations of 200 and 500 µM seemed to cause damage and impair the activities of PDL cells. Our results were similar to those of previous studies, which showed that PDL cells and gingival fibroblasts preferred low hypoxic conditions, which could enhance proliferation, osteogenic differentiation, 21 22 23 24 and type I collagen production 28 via HIF-1α in those cells. However, aged PDL cells have not yet been evaluated; our study concluded that a low concentration of H _2_ O _2_ may promote cellular activities, but not to the level observed in the young group. Due to oxygen deprivation caused by accumulated ROS, which frequently occurs during the aging process, the hypoxia-induced mechanism may be very active in aged cells. Therefore, the regeneration potential of aged cells could be comparable to that of younger cells.

To evaluate the aging process in PDL cells, a cellular senescent assay was initially performed in all groups. The senescence-associated marker β-galactosidase was detectable only in the aged group. β-galactosidase is a glycoside hydrolase enzyme that can catalyze the hydrolysis of β-galactosidase into monosaccharides only in senescent cells. Hence, the senescence of PDL cells from aged participants could be confirmed, but senescence was absent in the control and young groups.

In cytotoxicity assays, the release of LDH in the control, young, and aged groups was not significantly different among groups at all concentrations, but the results demonstrated a larger increase in cytotoxicity at 200 and 500 µM. Therefore, when compared to the cellular environment generated during aging processes, increasingly severe hypoxia in cells contributes to high ROS levels and is harmful to cells and their activities. The use of a cell therapy technique with cells from an aged subject should be the primary determinant of whether there is a low hypoxic condition, and the use of aged cells with a low level of hypoxia could be effective for periodontal regeneration. Cellular tolerance to H _2_ O _2_ varies by cell type and exposure time. Ji et al found that if the concentration of H _2_ O _2_ was not more than 25 µM when neuroblastic cells were exposed for 6 hours, it did not affect cell cytotoxicity. Meanwhile, 50, 100, and 200 µM concentrations induced cell death by 7, 15, and 16%, respectively, compared to the control. 29 Moreover, previous studies found that untreated endothelial cells or PDL cells can release LDH equal to 30 to 33% cytotoxicity at 24 hours. 30 31 LDH release from our study was approximately 8 to 30% with concentration-dependent trend, thus results were similar to those of a previous study that demonstrated the LDH release was time- and concentration-dependent. 32 Wang et al found that neuronal cells can tolerate H _2_ O _2_ concentrations up to 100 µM for 48 hours, 33 and Potier et al demonstrated that the cell cytotoxicity of mesenchymal cells is clearly decreased after 120 hours of hypoxic culture. 34 Thus, tolerance to oxygen-deprived conditions is dependent on the cells and the duration of the experiments.

Cellular migration of PDL cells at 25 and 50 µM were the appropriate concentrations that tended to promote *in vitro* wound closure. The young and aged groups exhibited a similar percentage of wound closure, which implied that aged and young PDL cells under low levels of hypoxia could be used and that they had similar tendencies during wound closure. Aziz et al demonstrated that concentrations of H _2_ O _2_ at 6.25 and 50 µM are the best concentrations to promote cell migration at 24 and 48 hours. 35 On the other hand, Iida et al found that hypoxic conditions enhanced the proliferation of human dental pulp cells if they were exposed to hypoxic conditions for more than 2 days. 36 This evidence indicated that a longer exposure period may enhance the cellular migration potential more than a shorter period. However, this study examined cultured PDL cells for only 2 days because long-term culture may conceal cellular migration instead of cell proliferation. Moreover, using H _2_ O _2_ -treated cells may induce more cell detachment, especially at high concentrations; thus, the longer exposure time may be a limitation for the cellular migration assay.

According to osteogenic differentiation, mineralization usually starts at approximately 7 days. Hence, we performed ARS at 7 and 14 days to detect mineralization. Our ARS results and their quantification demonstrated that mineralization at both 7 and 14 days was not different between the young group and the aged group. When analyzing osteogenic gene expression, RUNX2, early markers of osteogenesis and differentiation, showed their highest expression at 25 to 50 µM on day 7 in both aged and young groups. RUNX2 is highly expressed at 7 days and gradually decreases in expression at 14 days. 37 At 14 days, the aged group showed higher mineralization, as indicated by ARS, than the young group; according to Grynpas, mineralization shifts toward higher mineralization during aging. 38 In addition, the correlation of idiopathic osteosclerosis of the jaw and carotid artery calcification is related to increasing age. 39 However, even though the aged group exhibited high mineralization by ARS, RUNX2 and ALP expression levels in the young group were higher than those in the aged group. Although the concentration of H _2_ O _2_ was not significantly affected when osteogenic differentiation was induced, the results of all osteogenic genes showed that the most suitable concentration was 25 to 50 µM. OPN is highly expressed at 14 to 21 days as an early to intermediate marker of osteogenic differentiation. Both the young group and the aged group showed the same tendency, with a very clear OPN expression at 14 days in both groups. Moreover, Hung et al found that a short period of treatment with hypoxic conditions did not clearly enhance the osteogenic differentiation potential and other evidence suggested that differentiation was promoted under long-term hypoxia. 21 40 This situation resembles osteogenic differentiation in bone marrow, which lacks oxygen, and is appropriate for osteogenic differentiation of bone marrow stem cells. 41 42

To investigate the expression of genes related to hypoxic conditions and osteogenic differentiation, the expression of HIF-1α was detected. HIF-1α, a transcription factor, is induced under hypoxic conditions. Under normoxic conditions, the production of HIF is proportional to its breakdown, which maintains small amounts of HIF-1α within the cells. However, under hypoxic conditions, HIF-1α levels are increased within the cell. Thus, HIF-1α was suggested to be a linking factor that may play a key role in inducing the overexpression of angiogenesis-related factors and contribute to osteogenic differentiation. 21 22 23 24 43

In our experiment, hypoxia chemically induced by H _2_ O _2_ had different effects at different concentrations. Concentrations of 25 and 50 µM were potentially appropriate for cellular migration and osteogenic differentiation. Although the trend was not statistically significant, our results suggested that a low level of hypoxia may enhance the potential for cellular migration and osteogenic differentiation. Additionally, the aging process, which is always prone to hypoxic conditions, was examined in this study, and it was discovered that PDL cells from aged participants might have been used for osteogenic differentiation for periodontal treatment by periodontal regeneration. Thus, periodontal regeneration in aged individuals could be further investigated to identify approaches for regenerative cell-based therapy. Further study is needed for *in vivo* analysis and investigations of HIF-1α-related mechanisms should be performed in aged PDL cells.

## Conclusion

Low concentrations of hypoxic conditions could potentially promote the cellular migration and osteogenic differentiation of aged PDL cells comparable to younger cells, revealing a novel opportunity for using aged PDL cells from an aged individual when performing cell-based therapy for periodontal regeneration. Aging is not a contraindication for using autogenous cells sources.
