# Supplementary material for: Influence of Hypoxic Condition on Cytotoxicity, Cellular Migration, and Osteogenic Differentiation Potential of Aged Periodontal Ligament Cells
Source: Eur J Dent. 2024 May 17;19(1):70–9. doi: 10.1055/s-0044-1786844 (PMC11750308; doi:10.1055/s-0044-1786844)
Supplement: Supplementary file 1 — Supplementary Material [file 10-1055-s-0044-1786844-s23113225.pdf]

Supplementary Table S1 Primers used for real-time RT-PCR

Gene	Accession no.	Sequence	Product size (bp)
HIF-1α	NM_0243591	(F) 5′-CCCTACTATGTCGCTTTCTTGG-3′<br>(R) 5′-GTTTCTGCTGCCTTGTATGGG-3′	199
RUNX2	NlM_004348	(F) 5′-CCCCACGACAACCGCACCA T-3′<br>(R) 5′-GTCCACTCCG GCCCACA.AATC-3′	292
Alkaline phosphatase	NM_001127501	(F) 5′-AACATCAGGGACATTGACGTG-3′<br>(R) 5′-GTATCTCGGTTTGAAGCTCTTCC-3′	159
Osteopontin	NM_000582	(F) 5′-CTCCA.TT GACTCGAA.CGACTC-3′<br>(R) 5′-CAGGTCTGCGAA.ACTTCTTAGAT-3′	230
β-actin	NM_0011013	(F) 5′-AGAGCTACGAGCTGCC TGAC-3′<br>(R) 5′-AGCACTGTGTTGGCGTACAG-3′	184

Abbreviations: β-actin, beta-actin; bp, base pair; HIF-1α, hypoxia-induced factor-1 alpha; RT-PCR, reverse transcription polymerase chain reaction; RUNX2, runt-related transcription factor 2.

 © 2024. The Author(s).
